# The predicted σ^54^-dependent regulator EtpR is essential for expression of genes for anaerobic *p*-ethylphenol and *p*-hydroxyacetophenone degradation in “*Aromatoleum aromaticum*” EbN1

**DOI:** 10.1186/s12866-015-0571-9

**Published:** 2015-11-02

**Authors:** Imke Büsing, Mirjam Kant, Marvin Dörries, Lars Wöhlbrand, Ralf Rabus

**Affiliations:** Institute for Chemistry and Biology of the Marine Environment (ICBM), Carl von Ossietzky University Oldenburg, Oldenburg, Germany; Max Planck Institute for Marine Microbiology, Bremen, Germany

**Keywords:** σ^54^-Dependent regulator, Aromatic compounds, *p*-Ethylphenol, *p*-Hydroxyacetophenone, Anaerobic degradation, "*Aromatoleum aromaticum*" EbN1, *In frame* deletion mutation, Proteomics, Gene expression, Sequence comparison

## Abstract

**Background:**

The denitrifying betaproteobacterium "*Aromatoleum aromaticum*" EbN1 anaerobically utilizes a multitude of aromatic compounds via specific peripheral degradation routes. Compound-specific formation of these catabolic modules is assumed to be mediated by specific transcriptional activators. In case of the recently elucidated *p*-ethylphenol/*p*-hydroxyacetophenone pathway, the highly substrate-specific regulation was implicated to involve the predicted σ^54^-dependent, NtrC-type regulator EbA324. The latter was suggested to control the expression of the two neighboring gene clusters encoding the catabolic enzymes as well as a corresponding putative solvent efflux system. In the present study, a molecular genetic approach was used to study the predicted function of EbA324.

**Results:**

An unmarked *in frame* Δ*ebA324* (here renamed as Δ*etpR; p*-ethylphenol regulator) deletion mutation was generated. The Δ*etpR* mutant was unable to grow anaerobically with either *p*-ethylphenol or *p*-hydroxyacetophenone. Growth similar to the wild type was restored in the Δ*etpR* mutant background by *in trans* expression of plasmid-born *etpR*. Furthermore, expression of the "*p*-ethylphenol" gene clusters as well as corresponding protein formation was shown to depend on the presence of both, EtpR and either *p-*ethylphenol or *p*-hydroxyacetophenone. In the wild type, the *etpR* gene appears to be constitutively expressed and its expression level not to be modulated upon effector presence. Comparison with the regulatory domains of known phenol- and alkylbenzene-responsive NtrC-type regulators of *Pseudomonas* spp. and *Thauera aromatica* allowed identifying >60 amino acid residues in the regulatory domain (in particular positions 149 to 192 of EtpR) that may contribute to the effector specificity viz. presumptively restricted effector spectrum of EtpR.

**Conclusions:**

This study provides experimental evidence for the genome predicted σ^54^-dependent regulator EtpR (formerly EbA324) of "*A. aromaticum*" EbN1 to be responsive to *p*-ethylphenol, as well as its degradation intermediate *p*-hydroxyacetophenone, and to control the expression of genes involved in the anaerobic degradation of these two aromatic growth substrates. Overall, the presented results advance our understanding on the regulation of anaerobic aromatic compound catabolism, foremost based on the sensory discrimination of structurally similar substrates.

**Electronic supplementary material:**

The online version of this article (doi:10.1186/s12866-015-0571-9) contains supplementary material, which is available to authorized users.

## Background

The "*Aromatoleum*"/*Thauera*/*Azoarcus* cluster within *Betaproteobacteria* comprises most of the currently known denitrifiers capable of anaerobic degradation of aromatic compounds [1, 2]. "*Aromatoleum aromaticum*" EbN1 is a metabolically versatile and the first genome-sequenced representative of this cluster, completely oxidizing >20 different aromatic compounds under anoxic conditions. These include the alkylbenzenes toluene and ethylbenzene, phenol, as well as the alkylphenols *p*-cresol and *p*-ethylphenol [3–5]. The multiple sensory/regulatory proteins predicted from the genome were suggested to constitute a fine-tuned regulatory network [6]. Subsequent experimental studies indeed implicated the latter in substrate-specific formation of catabolic modules [4, 5, 7, 8], as well as in the adaptation to substrate-limiting [9] and stress conditions [10]. Synthesis of the hitherto accomplished physiological-proteomic insights qualifies "*A. aromaticum*" EbN1 as a promising systems biology model [11].

**Fig. 1:**
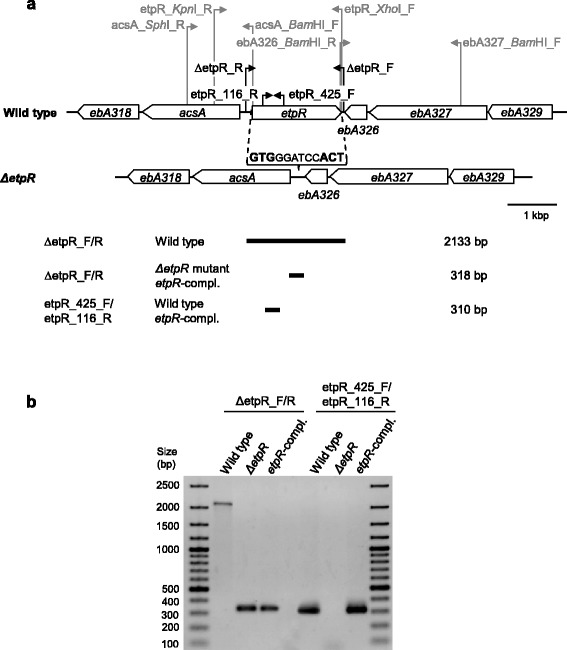
Generation of Δ*etpR* deletion and *etpR*-complemented mutants. Scale model of enlarged section of the "*p*-ethylphenol" gene clusters on the chromosome of "*A. aromaticum*" EbN1, displaying *etpR* and its 3'- and 5'-neighbouring regions in the wild type (top) and the Δ*etpR* mutant (bottom). The chromosomal hybridization location of primer pairs used for construction of the knockout vector pK19 Ω*acsAebA326/7* and the complementation plasmid pBBR1MCS-2 Ω*etpR* are shown in grey (Tables 2 and 3). The positions of the primer pair Δ*etpR*_F/R for identifying the knockout genotype and an *etpR*-specific primer pair are indicated in black and the lengths of the corresponding PCR products are given below the gene model (**a**). Electropherogram of PCR products obtained from the wild type, the Δ*etpR* mutant and the *etpR-*complemented mutant applying different primer pairs. Using the primer pair Δ*etpR*_F/R, a 2,133 bp long amplicon was obtained for the chromosome of the wild type and a shorter, 318 bp long, amplicon for the Δ*etpR* mutant and the *etpR-*complemented mutant. Accordingly, amplification of *etpR* (310 bp) was not possible for the Δ*etpR* mutant, but for the *etpR-*complemented mutant (**b**). Abbreviations: Δ*etpR*, Δ*etpR* mutant; *etpR-*compl., *etpR-*complemented mutant

The anaerobic degradation of the growth substrates *p*-ethylphenol and *p*-hydroxyacetophenone by "*A. aromaticum*" EbN1 was recently shown to be analogous to the ethylbenzene pathway [5]. Initial oxygen-independent hydroxylation to 1-(4-hydroxyphenyl)-ethanol and subsequent dehydrogenation to *p*-hydroxyacetophenone is followed by decarboxylation and thiolytic cleavage yielding *p*-hydroxybenzoyl-CoA. Proteins involved in this degradation pathway and a presumptively associated efflux system are encoded in a 15 kbp "catabolic" and a 6.5 kbp "efflux" gene cluster located in direct neighborhood on the chromosome of "*A. aromaticum*" EbN1. The specific transcriptional induction of these two "*p*-ethylphenol" gene clusters in the presence of *p*-ethylphenol as well as *p*-hydroxyacetophenone was previously inferred from the highly similar abundance profiles of respective transcripts and proteins [5]. This substrate-specific regulation was suggested to be mediated by the predicted σ^54^-dependent regulator EbA324 (here renamed as EtpR for *p*-ethylphenol regulator) that is encoded in between the "catabolic" and "efflux" gene clusters (Fig. 1a). Typically, σ^54^-dependent regulators consist of (i) an N-terminal regulatory, (ii) a central ATP-hydrolyzing (activating) and (iii) a C-terminal DNA-binding domain [12, 13]. Upon effector-binding, such regulators oligomerize and bind to distinct DNA-enhancer sequences upstream of the transcriptional start site [14]. In the process of transcription initiation, σ^54^ binds to a highly conserved −12/−24 consensus sequence (5'-TGGC-N_7_-TTGCA-3') and recruits RNA polymerase and the σ^54^-dependent regulator; the now formed RNA polymerase holoenzyme is activated by ATP-hydrolysis at the central domain of the regulator [15]. In accord, the promotor regions of the *p*-ethylphenol-related “catabolic” and “efflux” gene clusters in "*A. aromaticum*" EbN1 contain conserved σ^54^-DNA-binding motifs [5]. Regulation of aerobic aromatic compound degradation by σ^54^-dependent regulators has been well-studied in *Pseudomonas* spp. The NtrC-type regulators XylR and DmpR induce transcription of gene clusters for aerobic toluene/xylene [16] and phenolic compound degradation [17], respectively. In both cases, direct binding of the aromatic substrates or structurally related compounds to the regulatory domain relieves the repression of the ATPase activity of the central domain, allowing ATP-hydrolysis and subsequent transcription initiation [18, 19].

In the present study, an unmarked ∆*etpR in frame* deletion mutation was generated to verify the predicted regulatory function of EtpR. The ∆*etpR* mutant and the *in trans etpR-*complemented mutant were characterized by means of physiological experiments as well as on the molecular level.

## Results and discussion

### Generation of the ∆*etpR* and *etpR*-complemented mutants

The unmarked *in frame* ∆*etpR* deletion mutation was generated to test the predicted regulatory function of EtpR in mediating *p*-ethylphenol- and *p*-hydroxyacetophenone-specific expression of the two “*p*-ethylphenol” gene clusters. In the Δ*etpR* mutant, only the start and stop codons of *etpR* were preserved to maintain the reading frame (Fig. 1a). Accordingly, no PCR product could be obtained using *etpR*-specific primers and only a small 318 bp amplicon was observed applying primers targeting the up- and downstream intergenic regions of *etpR* (wild type amplicon 2,133 bp; Fig. 1b). In addition, correctness of the deletion site and its 5'- and 3'-flanking regions (i.e., ∆*etpR* genotype) was confirmed by nucleotide sequencing (Additional file 1: Figure S1). This newly generated ∆*etpR* mutant was *in trans* complemented via an *etpR*-bearing broad-host range plasmid, yielding constitutive expression of *etpR* (see below). The resultant *etpR*-complemented mutant had the genotype Δ*etpR*, pBBR1MCS-2 Ω*etpR*.

### The ∆*etpR* mutant cannot grow with *p-*ethylphenol and *p*-hydroxyacetophenone

Detailed growth analyses of the Δ*etpR* mutant were performed in comparison to the wild type and the *etpR*-complemented mutant*.* All three strains were benzoate-adapted and transferred at ½ OD_max_ to fresh media containing either benzoate, *p*-ethylphenol, *p*-hydroxyacetophenone or a binary mixture of benzoate and *p*-hydroxyacetophenone as sole source(s) of carbon and energy. Selected growth parameters are compiled in Table 1.

**Table 1:** Selected growth parameters determined for wild type, Δ*etpR* mutant and *etpR*-complemented mutant (*etpR*-compl.) of "*A. aromaticum*" EbN1^a^

			Consumption rate (mM h^−1^)		
Substrate(s)^b^	Genotype^d^	μ_max_ (h^−1^)	Substrate 1	Substrate 2	Lag-phase (h)
	wild type	0.121	0.35	-	10
Bz	Δ*etpR*	0.121	0,34	-	10
	*etpR*-compl.	0.130	0.40	-	9
	wild type	0.064	0.03	-	57
pHac	Δ*etpR*	-	-	-	-
	*etpR*-compl.	0.067	0.05	-	28
	wild type	0.093	0.07	-	29
pEp	Δ*etpR*	-	0.01	-	-
	*etpR*-compl.	0.098	0.08	-	20
	wild type	0.067	0.11	0.03	13
Bz^1^ + pHac^2c^	Δ*etpR*	0.082	0.14	-	13
	*etpR*-compl.	0.053	0.06	0.05	15
	wild type	0.091	0.14	0.08	14
Bz^1^ + pEp^2c^	Δ*etpR*	0.096	0.14	0.05	14
	*etpR*-compl.	0.135	0.12	0.08	16

^a^All growth experiments were performed in duplicates with high reproducibility; the given values are mean values

^b^Bz, benzoate; pHac, *p*-hydroxyacetophenone; pEp, *p*-ethylphenol

^c^Superscript “1” denotes substrate 1; superscript “2” denotes substrate 2

^d^Δ*etpR*, Δ*etpR* mutant; *etpR*-compl., *etpR*-complemented mutant

#### Growth with benzoate

**Fig. 2:**
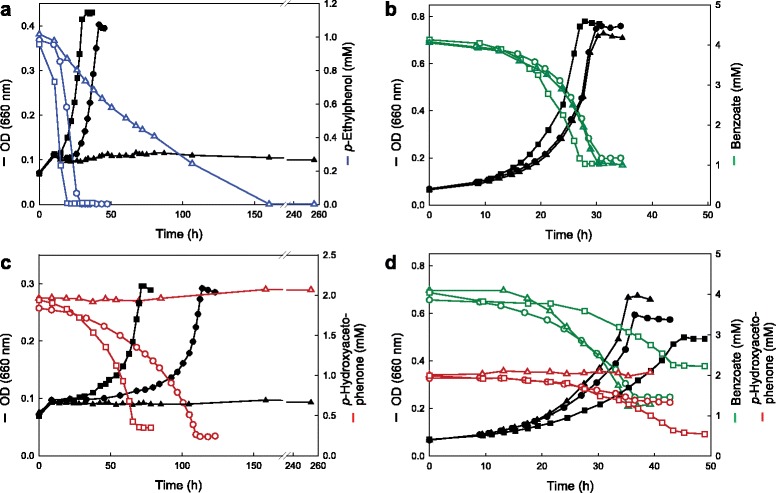
Physiological characterization of strains. Growth of "*A. aromaticum*" EbN1 wild type (circles), Δ*etpR* mutant (triangles) and *etpR-*complemented (genotype: Δ*etpR*, pBBR1MCS-2 Ω*etpR*) mutant (squares) under nitrate-reducing conditions with *p*-ethylphenol (blue, **a**), benzoate (green, **b**), *p*-hydroxyacetophenone (red, **c**) and a binary mixture of benzoate and *p*-hydroxyacetophenone (**d**). Cultures were inoculated with cells adapted to anaerobic growth with benzoate. All growth experiments were performed in duplicates with high reproducibility; for each experiment one representative experiment is depicted. The wild type harboring pBBR1MCS-2 showed wild type-like growth with *p*-hydroxyacetophenone. In contrast, the ∆*etpR* mutant with pBBR1MCS-2, like the ∆*etpR* mutant, was unable to utilize *p*-hydroxyacetophenone (data not shown). Optical density: black, filled symbols; substrate concentrations: colored, open symbols

Growth of the wild type and the Δ*etpR* mutant with benzoate was nearly identical, while the *etpR*-complemented mutant grew slightly faster, despite the presence of kanamycin in the medium (Fig. 2b). Furthermore, the Δ*etpR* mutant displayed growth similar to the wild type with all other known substrates (data not shown), except for *p*-ethylphenol and *p*-hydroxyacetophenone.

#### Growth with p-ethylphenol and p-hydroxyacetophenone

The Δ*etpR* mutant could not grow with *p*-ethylphenol and *p*-hydroxyacetophenone (Fig. 2a, c). The minute initial increase in optical density can probably be attributed to consumption of residual benzoate, co-transferred during initial inoculation. The unexpected complete depletion of *p*-ethylphenol from Δ*etpR* mutant cultures after ~160 h of incubation is most likely due to unspecific membrane accumulation, as it is not coupled to denitrification and also observable in wild type cultures with *de novo* protein synthesis inhibited by kanamycin (see Additional file 1: Figure S2). Noteworthy, the *etpR*-complemented mutant started to grow after a markedly shorter lag-phase as compared to the wild type, even though maximum growth rates and substrate depletion profiles of the two strains were similar (Fig. 2a, c; Table 1). This shorter lag-phase may be due to the higher EtpR abundance in the complemented strain (see below).

#### Growth with a binary mixture of benzoate and p-hydroxyacetophenone

When the three strains were shifted from benzoate to a binary mixture of benzoate and *p-*hydroxyacetophenone, highest rates of growth and benzoate consumption were observed for the Δ*etpR* mutant (Table 1), while the concentration of *p*-hydroxyacetophenone remained unchanged in this strain (Fig. 2d). However, growth rate and OD_max_ were lower as compared to growth with benzoate, most likely due to the toxicity of *p*-hydroxyacetophenone. Rates for growth and benzoate consumption of the wild type with the binary substrate mixture were higher as compared to the *etpR*-complemented mutant, but the rate of concomitant *p*-hydroxyacetophenone depletion was lower (Table 1). Hence, the *etpR*-complemented mutant seems to more preferentially consume *p*-hydroxyacetophenone than benzoate as compared to the wild type (Fig. 2d). Similar results were obtained for growth experiments with a binary mixture of benzoate and *p*-ethylphenol (Additional file 1: Figure S3).

### EtpR mediates substrate-specific expression of the "*p*-ethylphenol" gene clusters

The simultaneous utilization of benzoate and *p*-hydroxyacetophenone (see preceeding paragraph), i.e., absence of catabolite repression, is a prerequisite for subsequent analysis of transcript and proteomic profiles. In the ∆*etpR* mutant benzoate sustains growth while at the same time the hypothesized loss of response to the effector *p*-hydroxyacetophenone due to *etpR* deletion can be tested. *p*-Hydroxyacetophenone was used as substitute of *p*-ethylphenol due to (i) its higher water solubility (not requiring provision via a carrier phase), (ii) the apparent absence of a passive uptake as observed for *p*-ethylphenol (Fig. 2a, c) and (iii) the uniform induction of gene expression and protein formation by both substrates [5].

**Fig. 3:**
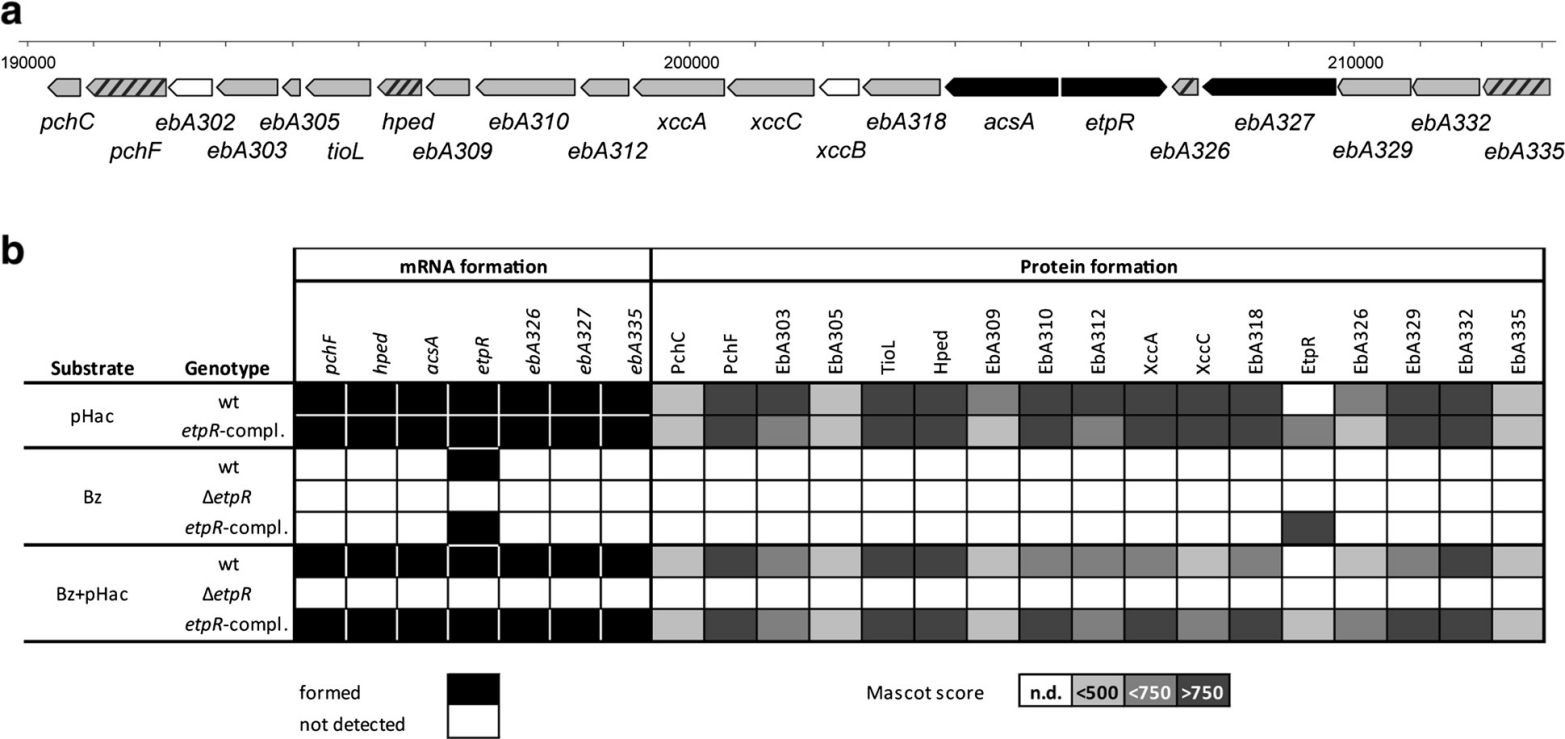
Transcript and proteomic analysis of the *p*-ethylphenol degradation pathway. Scale model of the chromosomal organisation of genes encoding proteins involved in the anaerobic degradation of *p*-ethylphenol and *p*-hydroxyacetophenone in "*A. aromaticum*" EbN1. Genes selected for transcript analyses are marked in black and identified proteins highlighted in grey (hatched if both applies) (**a**). Transcript and proteomic analysis of wild type, Δ*etpR* mutant and *etpR-*complemented (genotype: Δ*etpR*, pBBR1MCS-2 Ω*etpR*) mutant grown with either *p*-hydroxyacetophenone (pHac), benzoate (Bz) or a binary mixture of benzoate and *p*-hydroxyacetophenone (Bz + pHac). Detected transcripts are indicated in black. Mascot scores for identified proteins are indicated by the intensity of grey shading (lowest Mascot score 192; highest Mascot score 1528) (**b**). According to recent functional characterization, Hped was renamed from its original designation ChnA [47]

Gene expression was analyzed for the wild type, the Δ*etpR* mutant and the *etpR*-complemented mutant anaerobically grown with benzoate as single substrate or a binary mixture of benzoate and *p*-hydroxyacetophenone. As reference, transcripts were analyzed for *p*-hydroxyacetophenone-grown cells of the wild type and the *etpR*-complemented mutant. Target genes were located at the beginning, in the middle and at the end of the two "*p*-ethylphenol" gene clusters, i.e., *acsA*, *hped* and *pchF* for the "catabolic" gene cluster and *ebA335*, *ebA327* and *ebA326* for the "efflux" gene cluster (Fig. 3a). Transcripts of both gene clusters were only detected in wild type and *etpR*-complemented mutant cells growing with either *p*-hydroxyacetophenone or the binary substrate mixture (Fig. 3b). In accord, 16 out of the 24 proteins encoded in the two "*p*-ethylphenol" gene clusters were only detected in the corresponding subproteoms. In case of the Δ*etpR* mutant, none of these transcripts or proteins was detectable in cells grown with the binary substrate mixture (Fig. 3b). Similar proteomic results were obtained with a binary mixture of benzoate and *p*-ethylphenol (Additional file 1: Figure S3). Hence, expression of the “*p*-ethylphenol” gene clusters depends on the presence of EtpR and either *p*-ethylphenol or *p*-hydroxyacetophenone. The expression of genes framing the knockout locus (i.e., *ebA327*, *ebA326* and *acsA*) by the *etpR*-complemented mutant in the presence of *p*-hydroxyacetophenone confirms the absence of polar effects arising from *in frame* deletion of the *etpR* gene.

Non-detection of the EtpR protein in the wild type suggests protein abundance below the detection limit of the applied method. In contrast, EtpR was detected in all samples of the *etpR*-complemented mutant with high confidence indicating a markedly higher abundance of EtpR. The absence of "*p*-ethylphenol" gene cluster expression in benzoate-utilizing cells of the *etpR*-complemented mutant, despite the artificially high EtpR abundance in this strain, demonstrates the effector dependence of EtpR for transcriptional activation.

### Hints on constitutive expression of *etpR*

**Fig. 4:**
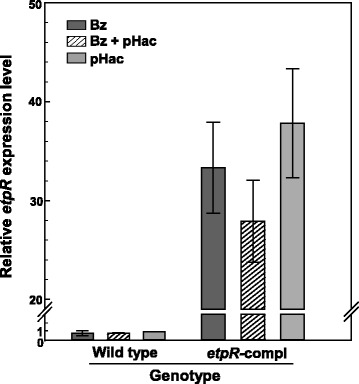
Analysis of *etpR* expression. Relative expression levels of *etpR* in the wild type and the *etpR-*complemented (genotype: Δ*etpR*, pBBR1MCS-2 Ω*etpR*) mutant grown with benzoate (Bz), *p*-hydroxyacetophenone (pHac) and a mixture of both substrates (Bz + pHac). Cells of the wild type adapted to growth with *p*-hydroxyacetophenone served as reference state and the *bcrC* gene (encoding the γ-subunit of benzoyl-CoA reductase) was used as reference gene

Binding of the XylR protein to the upstream region of the *xylR* gene was previously reported to reduce the σ^70^-dependent expression of the latter, by what XylR negatively influences its own formation; moreover, effector presence was shown to enhance this effect [16, 20, 21]. To investigate if a similar feedback mechanism exists for EtpR, transcription of *etpR* was quantitatively analyzed in the wild type and the *etpR*-complemented mutant grown with benzoate, *p*-hydroxyacetophenone and a binary mixture of benzoate and *p*-hydroxyacetophenone. The wild type displayed even expression levels (−1.2-fold; Fig. 4) independent of the growth substrate(s) and effector presence, respectively. If EtpR influenced its own formation as reported for XylR, i.e. stronger decrease of expression due to effector presence, a significantly higher *etpR* expression level would have to be expected for the wild type in presence of benzoate as compared to the *p*-hydroxyacetophenone(effector)-containing conditions. Conversely, a positive feedback may also be unlikely, as the *etpR* upstream region does not contain a −12/−24 consensus sequence. Taken together, expression of the *etpR* gene is apparently constitutive.

### Wild type EtpR abundance probably sufficient for full-level transcription

The *etpR*-complemented mutant strongly expressed *etpR* under all three tested substrate conditions (>28-fold *vs*. wild type; Fig. 4), agreeing with the medium copy number of the vector (around ten), the strong vector-inherent promoter, and the exclusive detection of the EtpR protein. Notably, the artificially high abundance of EtpR in the *etpR*-complemented mutant did not yield an increased abundance of proteins involved in *p*-ethylphenol degradation (at ½OD_max_) as compared to the wild type (Fig. 3b). Hence, maximum transcription/translation levels are apparently achieved also with the lower EtpR abundance occurring in the wild type, which is also reflected by the similar μ_max_ of both strains with *p*-ethylphenol or *p*-hydroxyacetophenone. The higher EtpR level in the *etpR*-complemented mutant may allow for reaching the maximum levels of catabolic proteins faster, which may explain the significantly shorter lag-phase as compared to the wild type (Fig. 2a, c; Table 1).

### Sequence comparison of regulatory domains

**Fig. 5:**
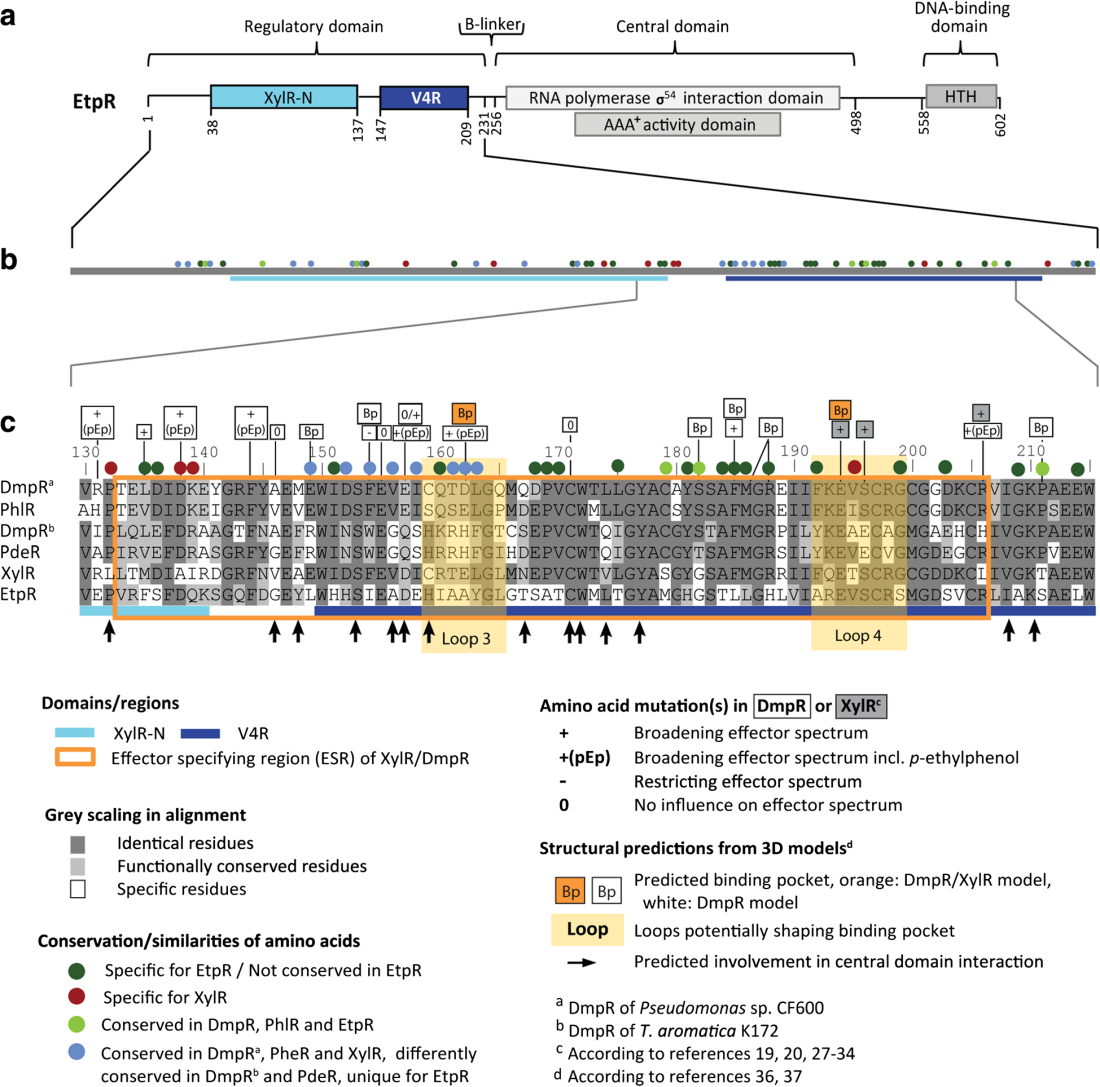
Amino acid sequence comparison of σ^54^-dependent regulators involved in aromatic compound catabolism. Domain structure of EtpR: XylR-N, activator of aromatic catabolism (IPR010523); V4R, 4-vinyl reductase (IPR004096); RNA-polymerase/σ^54^-interaction domain (IPR002078); AAA+ ATPase domain (IPR003593); DNA-binding HTH domain, Fis-type (IPR002197) (**a**). Schematic enlargement of the regulatory domain (bold grey line) displaying the positions of amino acid residues conserved/similar among the six compared regulatory proteins. **b** Sequence alignment of the effector specifying region (ESR) in the V4R domain of: *Pseudomonas* sp. DmpR (YP_009074430), PhlR (YP_009074867) and XylR (YP_009074187); *T. aromatica* K172 DmpR (CAC12684); "*A. aromaticum*" EbN1 PdeR (CAI07889) and EtpR (CAI06292) (**c**). The full-length alignment of the regulatory domain sequences is provided in the Additional file 1: Figure S5

EtpR belongs to the σ^54^-dependent NtrC/XylR-type transcriptional regulators with the typifying architecture of a less well conserved effector-binding N-terminal regulatory domain, connected to the more conserved central activating and C-terminal DNA-binding domains (Fig. 5a) [5, 12, 13].

#### Known phenol- or alkylbenzene-specific NtrC-type regulators

DmpR and PhlR are closely related regulators of aerobic phenol catabolism in *Pseudomonas* sp. CF600 [17, 22] and *P. putida* H [23], respectively. They are activated by direct binding of phenol or derivatives thereof, e.g., cresol and dimethylphenol isomers [24, 25] (Additional file 1: Figure S4). Notably, DmpR also recognizes *o*- and *m*-ethylphenol [25]. Similarly, DmpR of *Thauera aromatica* K172 [26] and PdeR of "*A. aromaticum*" EbN1 [4, 6] are suggested to regulate anaerobic phenol degradation; differential proteomics indicated PdeR to also respond to *p*-cresol [4]. XylR of *P. putida* regulates aerobic toluene degradation, and is activated by this as well as other alkylbenzenes such as *m*- and *p*-xylene [16].

Alteration of regulatory domain residues in XylR and DmpR from *Pseudomonas* spp. yielded broadened or restricted effector spectra, or even completely inhibited the response to aromatic effectors [19, 20, 27–34]. Most of these residues are confined to a stretch of 75 amino acids, defined as effector specifying region (ESR) [35]. 3D-models of XylR and DmpR predicted structural features for shaping an effector-binding pocket and interaction with the central domain [36, 37]. The current knowledge on the regulatory domain is compiled in Additional file 1: Figure S5.

#### Comparison of EtpR to known regulators

The phylogeny of the inspected regulatory domains reflects effector spectra as well as deployment for anaerobic *vs*. aerobic catabolism. EtpR branches off from the "anaerobic" phenol-specific regulators (PdeR of "*A. aromaticum*" EbN1 and DmpR of *T. aromatica* K172), while the three together separate from those of aerobic phenol degradation (DmpR and PhlR of *Pseudomonas* spp.). The alkylbenzene-specific XylR of *P. putida* is more distantly related to the other five regulators (Additional file 1: Figure S4).

Current proteomic data demonstrated specific formation of the "*p*-ethylphenol"-proteins only in cells anaerobically growing with *p*-ethylphenol or *p*-hydroxyacetophenone, but not with 20 other aromatic substrates [5, 11]. Therefore, activation of EtpR may be restricted to the former two. Such a narrow effector spectrum would distinct EtpR from the above described three regulators of *Pseudomonas* spp..

To identify primary sequence features, possibly linked to the effector spectrum of EtpR, aligned regulatory domain sequences were analyzed (Additional file 1: Figure S5), with the ESR emphasized in Fig. 5 (amino acid positions 130–205 in EtpR). Across these six complete regulatory domains, 43 residues (20.7 %) were strictly conserved, comprising most of the reported conserved residues of NtrC-type regulators [36]. Sixty-two other residues (29.8 %; colored circles) are conserved to differing degrees and are mostly ESR-located; they may therefore be effector specifying for EtpR and are briefly described in the following.

Recognition of the phenolic moiety may involve 8 residues (4 in the ESR; brown circles) that are exclusively conserved in the five phenolic compound-sensing regulators (incl. EtpR). Further 6 residues (light green circles) could also contribute to phenol-specificity, as they are conserved in DmpR, PhlR and PdeR, but not in alkylbenzene-sensing XylR and alkylphenol-sensing EtpR.

Notably, a total of 28 residues (dark green circles) are conserved in all proteins except for EtpR, with 16 of them located in the ESR. The sensory relevance of these residues is reflected by four of them being located in the predicted binding pocket of DmpR and XylR [36] and two at locations that broaden the effector spectrum of DmpR [30, 31]. From an inversed perspective, these 28 residues are distinct in EtpR. They may therefore be involved in specific sensing of, e.g., the keto group in *p*-hydroxyacetophenone, since the latter is not known to be an effector of the other five compared regulators. Alternatively, these 28 residues may at least indirectly contribute to a structural shaping of the EtpR-specific sensory properties. These scenarios may also account for another group of 20 residues (blue circles), which are differently conserved in DmpR, XylR and PhlR of *Pseudomonas* spp. as compared to DmpR of *T. aromatica* and PdeR of "*A. aromaticum*" EbN1, but are again distinct in EtpR.

More than 17 residue changes (marked by a "+" sign) in the regulatory domains of *Pseudomonas* DmpR and XylR (13 in the ESR or close by) broaden their effector spectra (partly also towards *p*-ethylphenol) [19, 31, 34]. They may not be effector specifying for EtpR, as none of the changes yields EtpR-residues.

Notably, the effector specificity of EtpR may not only be attributed to single residues, since also the interaction of different regions contributes to defining effector spectra, i.e., loops (yellow areas) and interactions of regulatory and central domain (black arrows) [36].

## Conclusions

Aerobic aromatic compound degradation pathways of *Pseudomonas* spp. may accommodate several, structurally related aromatic substrates, e.g., toluene, *m*- and *p*-xylene as well as 1,2,4-trimethylbenzene in case of *P. putida* [21]. This substrate promiscuity of single pathways is also reflected in rather broad effector spectra of the involved transcriptional regulators [16, 25]. In contrast, the substrate ranges of the individual anaerobic pathways in "*A. aromaticum*" EbN1 appear to be more restricted and the substrate-specific expression of their genes is assumed to be individually controlled by corresponding one- or two-component regulatory systems [11]. The observed highly *p*-ethylphenol/*p*-hydroxyacetophenone-specific induction of the anaerobic *p*-ethylphenol degradation pathway by EtpR can be attributed to a concurrence of unique sets of amino acids accumulating in the ESR (accounting for 36.8 %), in particular between position 149 and 192. Since they are most specific for EtpR as compared to the other regulators, they possibly specify the sensing of phenolic-, ketonic- and/or alkylbenzylic-moieties, and determine the observed narrow effector spectrum of EtpR. This further supports a general role of ESRs in mediating sensory recognition of aromatic compounds as previously reported for DmpR and XylR of *Pseudomonas* spp. [35]. The apparent constitutive, low level expression of *etpR* in the wild type allows for a full level formation of catabolic proteins that may be only reached faster at higher regulator abundance as observed for the *etpR*-complemented mutant. Hence, the extent of "*p*-ethylphenol" gene cluster expression should depend on the presence of the substrates/effectors *p*-ethylphenol and *p*-hydroxyacetophenone rather than on the abundance of the regulator itself (EtpR). Overall, the present study demonstrates EtpR (EbA324) to serve as transcriptional regulator in the *p*-ethylphenol catabolism, and represents to our knowledge the first molecular genetic study on a σ^54^-dependent regulator in an anaerobic aromatic compound degrader.

## Methods

### Bacterial strains and cultivation

"*A. aromaticum*" EbN1 and *E. coli* S17-1 were cultivated as described previously [3, 38]. Strains used and generated in this study are summarized in Table 2.

**Table 2:** Strains and plasmids used in this study

	Genotype and/or characteristics	Reference
Strains		
“*Aromatoleum aromaticum*” EbN1	Wild type	[3]
EbN1*-*RR001 (Δ*etpR* mutant)	Δ*etpR*	This study
EbN1*-*RR002 (*etpR*-complemented mutant)	Δ*etpR*, pBBR1MCS-2 Ω*etpR*	This study
EbN1-RR003 (Wild type containing pBBR1MCS-2)	Wild type, pBBR1MCS-2	This study
EbN1-RR004 (Δ*etpR* mutant containing pBBR1MCS-2)	Δ*etpR,* pBBR1MCS-2	This study
* Escherichia coli* S17-1	*Pro, thi, hsd*R, *Tra* ^+^, *rec*A^−^, Tr^r^, Sm^r^, ΩRP4-TE::Mu-Kn::Tn7	[45]
Plasmids		
pK19*mobsacB*	Km^R^, *sacB* modified from *B. subtilis*, *lacZα*	[46]
pK19 Ω*acsA*	Km^R^, *sacB* modified from *B. subtilis*, *lacZα*, *acsA* from "*A. aromaticum*" EbN1	This study
pK19 Ω*acsAebA326/7*	Km^R^, *sacB* modified from *B. subtilis*, *lacZα*; *acsA*, *ebA326* and part of *ebA327* from "*A. aromaticum*" EbN1	This study
pBBR1MCS-2	Km^R^, *mob, lacZα*	[40]
pBBR1MCS-2 Ω*etpR*	Km^R^, *mob, lacZα, etpR* from "*A. aromaticum*" EbN1	This study

### Generation of the *in frame* ∆*etpR* deletion mutation

Genomic DNA and plasmids were isolated according to standard methods [39]. Oligonucleotide primers were designed using the Lasergene software (version 7.0; DNASTAR, Madison, WI, USA) and purchased from Biomers GmbH (Ulm, Germany). Nucleotide sequences of applied primers and primer details are provided in Table 3.

**Table 3:** Oligonucleotide primers applied in this study

Primer^a^	Target gene	Nucleotide sequence (5' → 3')^b^	Product length (bp)
**Gene specific primer pairs**			
bcrC_108_F	*bcrC*	CAAGTGGTGGCAACGATGTGT	191
bcrC_299_R		GAAGGTCTGGCGATACTGGATGC	
pchF_1336_F	*pchF*	GGCCGGCAACGTCATCATC	273
pchF_1099_R		CCATCCGGGAGCACCACT	
hped_153_F	*hped*	TGATCGAAGGCAAGGGCGGAAAAG	331
hped _473_R		GCGGCGGTGTAGGGCGTGATG	
acsA_1373_F	*acsA*	GCCGCGGTGAGGTT	306
acsA_1068_R		CGGGGTGAATGTCCA	
etpR_425_F	*etpR*	AATTGGCCGCTCTTCTG	310
etpR_116_R		TTTCGGCATGTTTGTCA	
etpR_1717_F^c^	*etpR*	TGGCGACGGCATTCTC	219
etpR_1499_R^c^		TGCCGCATCTGTTCACC	
ebA326_41_F	*ebA326*	TGGCTGGATCTCTGCTC	275
ebA326_315_R		TTCCCGTGCGACCTG	
ebA327_1070_F	*ebA327*	GCTTCGCGGTCCTGA	375
ebA327_1480_R		TGTCGCGGTTGTAGC	
ebA329_212_F	*ebA329*	TGCGGCCCCTGATG	316
ebA329_537_R		ACGATGCCGCTGTGG	
ebA332_488_F	*e*bA*332*	CCGGCGTGGAGGTAG	285
ebA332_772_R		GGCGCGGGGTTTT	
ebA335_1092_F	*ebA335*	GCTGGGGGAGACGAA	253
ebA335_1344_R		CGCCGCCTTGTTGT	
**Generation of Δ** ***etpR*** **deletion mutation**			
acsA_BamHI_F	*acsA*	AA**GGATCC**CACGAAATGTCTCCTGAACCCTGC	1300
acsA_SphI_R		ACCGG**GCATGC**GCCCACCAGC	
ebA327_BamHI_F	*ebA326/ebA327*	GATCA**GGATCC**ACGTCACCG	2350
ebA326_BamHI_R		AA**GGATCC**TGACCGTCGGAGGACCGGATAGATC	
**Identification of Δ** ***etpR*** **genotype**			
Δ*etpR* _F	3'-IR-*etpR* ^f^	TGGGCGTAGCGTAGT	2133^d^/ 318^e^
Δ*etpR* _R	5'-IR-*etpR* ^f^	TGGATTGTTCTGTAT	
**Generation of** ***in trans*** **complementation of** ***etpR***			
etpR_XhoI_F	*etpR*	AA**CTCGAG**CCCACTCCAAGCGTCGAAACACCGGC	2436
etpR_KpnI_R		AAA**GGTACC**GCTTCGCTCCGGGAAACCAGTGTGCGC	

^a^R = reverse primer; F = forward primer

^b^Recognition sites for restriction enzymes are marked in bold type

^c^Primer pair applied in real time RT-PCR experiment

^d^"*A. aromaticum*" EbN1 (wild type)

^e^Deletion mutant (strain EbN1-RR001; Δ*etpR* genotype)

^f^IR, intergenic region

For unmarked knockout of the *etpR* (*ebA324*) gene, a knockout vector based on the suicide vector pK19*mobsacB* [38] was constructed in an *E. coli* S17-1 background, containing 2.4 kbp of the 5'-region and 1.3 kbp of the 3'-region of *etpR*. Initially, the 5'-region containing parts of the *acsA* gene were cloned into pK19*mobsacB* using *Sph*I and *Bam*HI restriction sites as reported [38], yielding the plasmid pK19 Ω*acsA*. Subsequently, the 3'-region, containing *ebA326* and parts of the *ebA327* gene, was cloned into plasmid pK19 Ω*acsA* using the *Bam*HI restriction site, resulting in the pK19 Ω*acsAebA326/7* knockout vector (Table 2). In the final vector construct, the start and stop codons of *etpR* were maintained, separated by a 6 bp *Bam*HI restriction site ("**GTG**GGATCC**ACT**" blow up in Fig. 1a). Homologous regions were amplified by PCR from genomic DNA of "*A. aromaticum*" EbN1 using a high fidelity polymerase (Phusion®; ThermoFisher Scientific, Dreieich, Germany; Table 3). The correctness of nucleotide sequences was verified by sequence analysis as described before [38] and pK19 Ω*acsAebA326/7* was transferred by conjugation from the *E. coli* S17-1 donor strain to "*A. aromaticum*" EbN1 according to the protocol described previously [38]. Integration of the knockout vector (single-cross over) gave rise to kanamycin resistant colonies and was verified by PCR using a primer pair (∆*etpR*_F/R*)* targeting the up- and downstream intergenic regions of *etpR*, yielding two amplicons of 318 bp and 2.13 kbp, respectively (Table 3; Fig. 1). The second cross-over (i.e., removal of the plasmid) was induced by several transfers of the single cross-over mutant in liquid medium without kanamycin. Colonies capable of growing on sucrose-containing medium were screened using the same primer pair as described above to identify a ∆*etpR* genotype in the deletion mutant strain EbN1-RR001 (Fig. 1). The genotype was verified by sequencing.

### Complementation of *etpR in trans* into the ∆*etpR* deletion mutant

A complementation vector for *in trans* expression of *etpR* was generated in an *E. coli* S17-1 background, based on the broad-host range vector pBBR1MCS-2 [40]. A 2.4 kbp nucleotide sequence was amplified by PCR, containing *etpR* as well as 300 bp upstream of the *etpR* start codon to also include the ribosomal binding site (Table 3; Fig. 1). This amplicon was cloned into the pBBR1MCS-2 vector using *Kpn*I and *Xho*I restriction sites and verified by sequencing. The vector was transferred via conjugation to the ∆*etpR* mutant yielding the *etpR*-complemented mutant strain EbN1-RR002 (genotype: Δ*etpR*, pBBR1MCS-2 Ω*etpR*) (Table 2; Fig. 1). Conjugation via agar-plate mating, identification for positive clones on selective media and PCR-based verification were carried out as previously described [38]. For control experiments, the pBBR1MCS-2 vector without *etpR* was conjugationally transferred to the wild type strain and the Δ*etpR* mutant yielding strain EbN1-RR003 (genotype: wild type, pBBR1MCS-2) and strain EbN1-RR004 (genotype: Δ*etpR*, pBBR1MCS-2), respectively (Table 2).

### Growth experiments

Growth experiments with the wild type, the ∆*etpR* mutant and the *etpR*-complemented mutant were carried out to characterize the phenotype of the generated ∆*etpR* deletion mutation. All three strains were adapted to anaerobic growth with benzoate for at least five passages. Anaerobic cultivation was conducted under nitrate-limited conditions with 400 ml medium in 500 ml flat bottles, sealed with rubber stoppers. Pre-cultures were provided with 4 mM benzoate as growth substrate and cells transferred at half-maximal optical density (½ OD_max_) to fresh medium supplemented with either of the following substrates: (i) *p*-ethylphenol (0.5 % (w/v) in 14 ml of the inert carrier phase 2,2,4,4,6,8,8-heptamethylnonane (HMN)), (ii) *p*-hydroxyacetophenone (2 mM), (iii) benzoate (4 mM) and (iv) a binary mixture of benzoate (4 mM) and either *p*-hydroxyacetophenone (2 mM) or *p*-ethylphenol (0.5 % (w/v) in 10 ml HMN). In case of cultures with benzoate or a binary substrate mixture, the medium contained 10 mM nitrate to achieve higher cell densities; while with *p*-ethylphenol and *p*-hydroxyacetophenone provided as single substrate only 7 mM nitrate were added to the medium.

Growth until depletion of the electron acceptor nitrate was monitored by measuring the optical density at 660 nm (UV–vis Spectrometer 1240; Shimadzu, Duisburg, Germany) and analysing the substrate concentrations in the culture supernatants with an UltiMate 3000 RSLC system (ThermoFisher Scientific, Germering, Germany). The supernatants were diluted and acidified (pH 2.0, 6 % acetonitrile) prior to analysis. Separation of analytes was achieved with a Dionex Acclaim 120 reversed-phase separation column (250 mm length, 2.1 mm inner diameter, 5 μm bead size; ThermoFisher Scientific). The column was temperature controlled at 25 °C and operated with a non-linear gradient of acetonitrile (5–90 % (v/v), pH 2.8) as eluent at a flow rate of 0.5 ml min^−1^: 2 min at 5 %, 5 to 14 % in 1 min, 14 to 39 % in 10.5 min, 39 to 90 % in 3 min, and 3 min at 90 %. Retention times (compound-specific wavelengths in parentheses) were the following: *p*-ethylphenol, 16.6 min (220 nm); *p*-hydroxyacetophenone, 9.4 min (270 nm); and benzoate, 11.8 min (236 nm).

For selected samples nitrate concentrations were determined by means of an ICS 1100 ion chromatography system (ThermoFisher Scientific). Nitrite and nitrate were separated using an IonPac™ AG9-HC separation column (250 mm length, 4 mm inner diameter, 9 μm bead size; ThermoFisher Scientific) and detected at a wavelength of 210 nm. Separation was achieved with sodium carbonate (9 mM) as the eluent administered at a flow rate of 1 ml min^−1^. The retention times (detection limits in parentheses) were as follows: nitrite, 9.1 min (1 μM); nitrate, 13.3 min (1 μM).

To assess the possibility of a passive depletion of *p*-ethylphenol from the medium, benzoate-adapted cells of the wild type were pre-grown with benzoate and transferred to anoxic medium with *p*-ethylphenol as described before. After 15 h, growth was inhibited by the addition of 50 μg ml^−1^ kanamycin. The concentration of *p*-ethylphenol in the cultures was analyzed by the RSLC system as given above.

### Cultivation for transcript and proteomic analysis

For profiling of compound-specific transcripts and proteome signatures, substrate-adapted wild type, the ∆*etpR* mutant and the *etpR*-complemented mutant were grown anaerobically with either (i) *p*-hydroxyacetophenone, (ii) benzoate or (iii) a binary mixture of benzoate and *p*-hydroxyacetophenone as sole organic substrate(s). Cells were cultivated in 250 ml flat bottles with 200 ml medium and harvested during linear growth at ½ OD_max_ as described by Champion et al. [41]. For each strain and growth condition, three replicate cultures were harvested for transcript and proteomic analyses, respectively (in total 6 replicate cultures each). Cell pellets for transcript analysis were treated with RNAprotect® Bacteria Reagent (Qiagen GmbH, Hilden, Germany) according to the manufacturer’s instructions.

### Preparation of mRNA and reverse transcription (RT)-PCR

Preparation of total RNA was performed according to the protocol of Oelmüller et al. (22) using cells treated with RNAprotect® Bacteria Reagent. Complete removal of DNA from the RNA preparation was verified by PCR. The quality (i.e., integrity) of the isolated RNA was analysed with MOPS-gels according to standard protocols [39]. cDNA was generated from two independent RNA preparations per strain and substrate condition, respectively, using the antisense primer of the target genes (Table 3). Reverse transcription was performed with 2.5 μg RNA applying the RevertAid H Minus Reverse Transcriptase (ThermoFisher Scientific) according to the manufacturer’s instructions. cDNA was amplified by PCR using the PCR MasterMix (Promega, Mannheim, Germany). Depending on the PCR efficiency of the gene-specific primer pairs, 1.0 or 2.0 μl of cDNA preparation were used as template per 20 μl PCR experiment comprising 20 or 39 PCR cycles.

To exclude polar effects resulting from the *in frame ΔetpR* deletion mutation and to qualitatively study gene expression of the two "*p*-ethylphenol" gene clusters located up and downstream of *etpR*, transcripts representative of both of them (Fig. 3) were analysed. Target genes were chosen such that both the first and last genes as well as the middle of each of the two gene clusters were covered, i.e., *acsA*, *hped* and *pchF* for the "catabolic" gene cluster, and *ebA335*, *ebA327* and *ebA326* for the "efflux" gene cluster.

### Real-time RT-PCR

Relative expression levels of *etpR* in the wild type and the *eptR*-complemented mutant were determined by real-time RT-PCR. *BcrC* (encoding the catalytic γ-subunit of benzoyl-CoA reductase) was selected as reference gene since benzoyl-CoA is a common intermediate in anaerobic degradation of benzoate as well as *p*-hydroxyacetophenone, and since *bcrC* expression is not regulated under the two investigated substrate conditions [5]. cDNA was generated from three individual RNA preparations per strain and growth condition as described above. Real-time PCR was carried out as reported by Kühner et al. [7] using an iQ5 real-time PCR detection system (Bio-Rad, Munich, Germany) and a qPCR MasterMix Plus for SyBR green I with fluorescin (Eurogentec, Cologne, Germany). The correctness of obtained PCR products was verified by sequencing. Determination of PCR efficiencies was performed as described by Ramakers et al. [42] and relative expression levels were calculated according to Pfaffl et al. [43]. At least three replicates of each individual cDNA preparation were analysed (in total 18 qPCR experiments). The wild type grown with *p*-hydroxyacetophenone served as reference state for calculation of relative gene expression levels (Fig. 4).

### Proteomic analysis

Whole cell shotgun analysis of substrate-adapted cells was performed as described recently [44]. Essentially, tryptic peptides were separated by a nanoLC system (UltiMate3000 nanoRSLC; ThermoFisher Scientific) operated in a trap-column mode and equipped with a 25 cm separation column (C18, 2 μm bead size, 75 μm inner diameter; ThermoFisher Scientific), applying a 280 min linear acetonitrile gradient. The nanoLC eluent was continuously analyzed by an online-coupled ion-trap mass spectrometer (amaZon speed ETD; Bruker Daltonik GmbH, Bremen, Germany) using a captive spray ion source (Bruker Daltonik GmbH). Per full scan MS (mass range 400–1400 *m*/*z*), 20 MS/MS spectra of the most intense doubly (or more highly) charged ions were acquired applying subsequent precursor exclusion for 0.2 min. Protein identification was performed using the ProteinScape platform (version 3.1; Bruker Daltonik GmbH) on an in-house Mascot server (version 2.3; Matrix Science Ltd, London, UK) based on the genome sequence of "*A. aromaticum*" EbN1 [6] and applying a target-decoy strategy as described [44]. Search results of the three biological replicates per test state were compiled and only proteins identified by at least 2 peptides were considered.

## Additional file

Additional file 1: Figure S1. Sequence verification of the ∆*etpR* mutant. **Figure S2**. Anaerobic growth with *p*-ethylphenol. **Figure S3**. Anaerobic growth with a mixture of benzoate and *p*-ethylphenol. **Figure S4**. Phylogenetic relationship of the regulatory domains of σ^54^-dependent NtrC-type regulators. **Figure S5**. Amino acid sequence comparison of σ^54^-dependent regulators involved in aromatic compound catabolism. (PDF 1242 kb)
