# Supplementary material for: The predicted σ54-dependent regulator EtpR is essential for expression of genes for anaerobic p-ethylphenol and p-hydroxyacetophenone degradation in “Aromatoleum aromaticum” EbN1
Source: BMC Microbiol. 2015 Nov 2;15:251. doi: 10.1186/s12866-015-0571-9 (PMC4630880; doi:10.1186/s12866-015-0571-9)
Supplement: Additional file 1: Figure S1. — Sequence verification of the ∆etpR mutant. Figure S2. Anaerobic growth with p-ethylphenol. Figure S3. Anaerobic growth with a mixture of benzoate and p-ethylphenol. Figure S4. Phylogenetic relationship of the regulatory domains of σ54-dependent NtrC-type regulators. Figure S5. Amino acid sequence comparison of σ54-dependent regulators involved in aromatic compound catabolism. (PDF 1242 kb) [file 12866_2015_571_MOESM1_ESM.pdf]

ADDITIONAL FILE 1

The predicted σ^{54}-dependent regulator EtpR is essential for expression of genes for anaerobic *p*-ethylphenol and *p*-hydroxyacetophenone degradation in "*Aromatoleum aromaticum*" EbN1

Imke Büsing, Mirjam Kant, Marvin Dörries, Lars Wöhlbrand and Ralf Rabus

Table of Contents

Figure S1 Sequence verification of the Δ*etpR* mutant……….……………………………….…...…………2

Figure S2 Anaerobic growth with *p*-ethylphenol..……………………………………………...…………3

Figure S3 Anaerobic growth with benzoate/*p*-ethylphenol mixture…….………….......………………..……4

Figure S4 Phylogenetic relations of regulatory proteins……………………………………..…………..6

Figure S5 Full length EtpR regulatory domain alignment…….………...………………………..…...…7

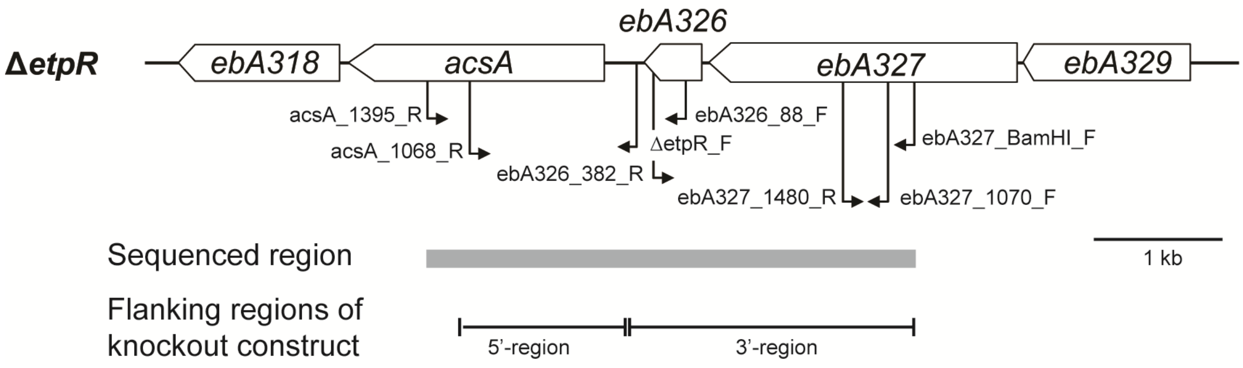

Primer[a]	Nucleotide sequence (5' → 3')[b]
acsA_1395_R	GATGACCGGTTCGCCCTGCTCGTT
ΔetpR _F	TGGGCGTAGCGTAGT
acsA_1068_R	CGGGGTGAATGTCCA
ebA326_88_F	GGCGCCCGAATAAC
ebA326_382_R	GGCGAGTGGGTCAGC
ebA327_1070_F	GCTTCGCGGTCCTGA
ebA327_1480_R	TGTCGCGGTTGTAGC
ebA327_BamHI_F	GATCAGGATCCACGTCACCG

Figure S1 Sequence verification of the Δ*etpR* mutant. Sequenced genomic region (ca. 4 kb; grey bar) of the Δ*etpR* mutant including the knockout locus and the 5'- and 3'-flanking regions of the knockout construct pK19 Ω*acsAebA326/7* (Table 2 and 3). Except for the deletion site, the sequenced region is identical to the wild type, demonstrating the absence of undesired cross overs and mutations in this region. The primer pairs used for sequencing are indicated and primer sequences are given in the table.

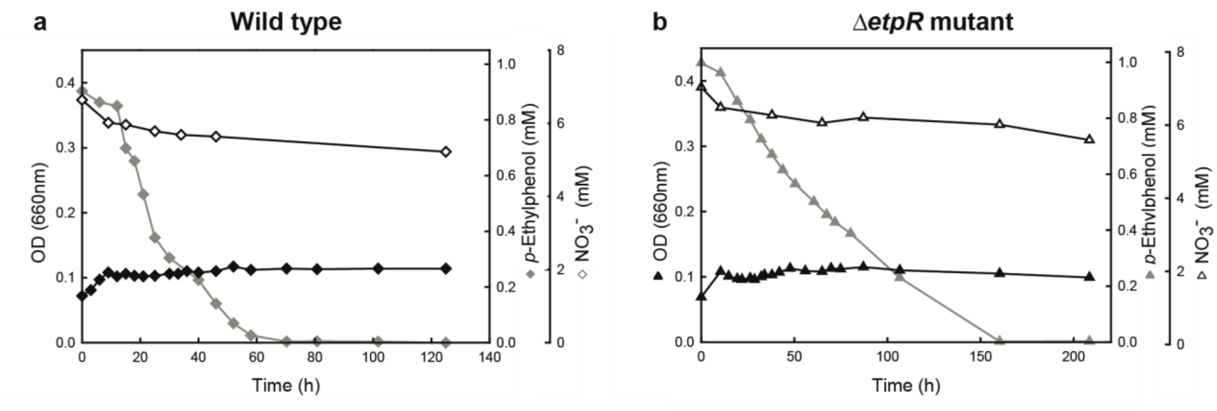

Figure S2 Anaerobic growth with *p*-ethylphenol. "*A. aromaticum*" EbN1 wild type and the Δ*etpR* mutant were grown with *p*-ethylphenol under nitrate-reducing conditions. Cultures were inoculated with cells adapted to anaerobic growth with benzoate. To analyze the observed passive *p*-ethylphenol uptake, 50 µg ml^{-1} kanamycin were added to the culture 15 h after inoculation at OD 0.12 when residual benzoate (transferred from inoculation) was consumed. Although growth of the culture stopped according to optical density measurements and only 1.4 mM nitrate was consumed within 110 h after kanamycin addition, *p*-ethylphenol was not detectable in the medium after 70 h (**a**). (B) In case of the Δ*etpR* mutant, *p*-ethylphenol depletion was slower. Removal from the medium took about 160 h with a concomitant consumption of 0.5 mM nitrate (**b**). Optical density: black, filled symbols; *p*-ethylphenol concentrations: grey symbols; nitrate concentrations: black, open symbols.

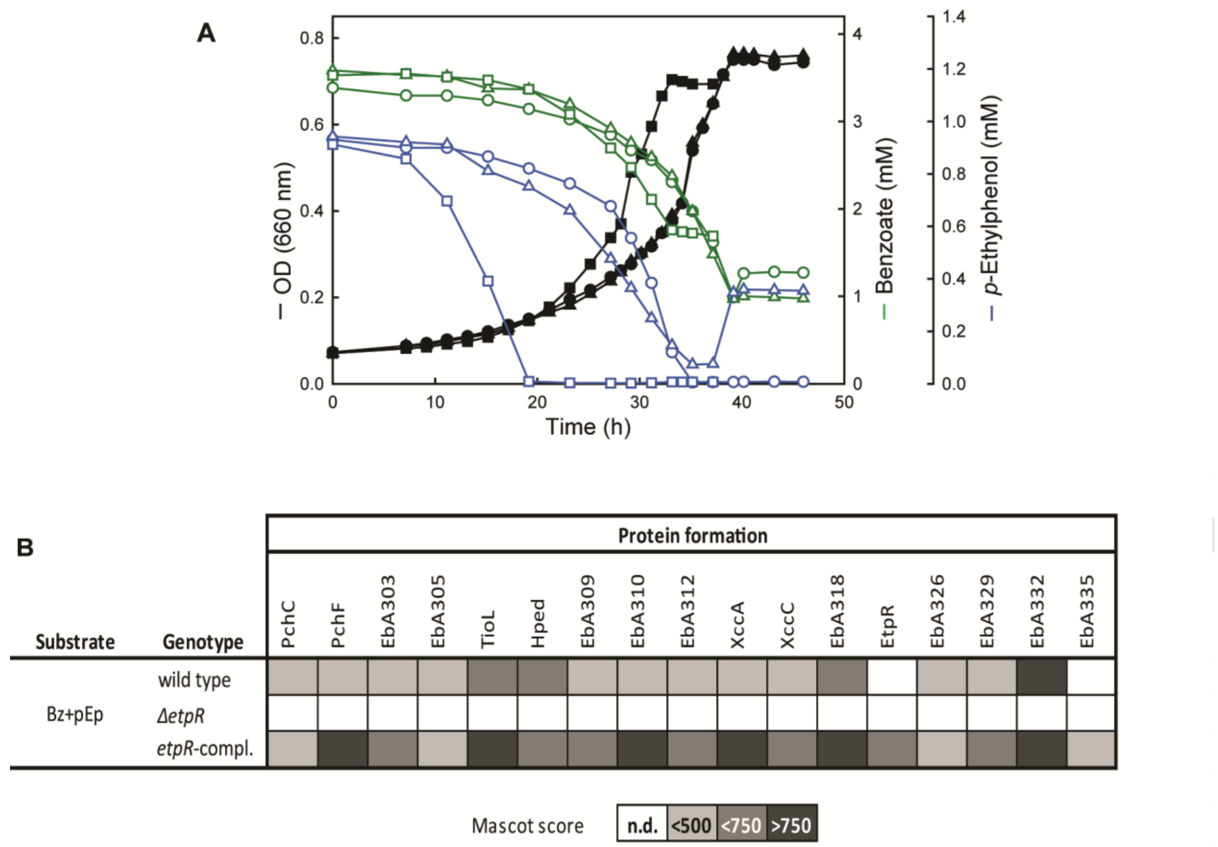

Figure S3 Anaerobic growth with mixture of benzoate and *p*-ethylphenol. Growth of "*A. aromaticum*" EbN1 wild type (circles), the Δ*etpR* mutant (triangle) and the *etpR*-complemented (genotype: Δ*etpR*, pBBR1MCS-2 Ω*etpR*) mutant (squares) under nitrate-reducing conditions with a mixture of benzoate (green) and *p*-ethylphenol (blue). Benzoate-adapted cells were shifted to the binary substrate mixture. Even though growth of the wild type and Δ*etpR* mutant was comparable, and growth and benzoate consumption rates differed only slightly (wt: 0.091 h^{-1} and 0.14 mM h^{-1}; Δ*etpR*: 0.096 h^{-1} and 0.14 mM h^{-1}), *p*-ethylphenol removal from the medium was different. While the time-course of *p*-ethylphenol concentration in the wild type resembles a typical substrate depletion profile with a rate of 0.08 mM h^{-1}, the *p*-ethylphenol depletion by the Δ*etpR* mutant was rather linear with a lower rate of 0.05 mM h^{-1}. Since *p*-ethylphenol was not completely removed from the medium by the Δ*etpR* mutant and its concentration increased again to >30% of the initial concentration (0.35 mM of initially 0.95

mM) after nitrate depletion, the assumption of a passive uptake mechanism is supported. The highest growth rate was observed for the *etpR*-complemented mutant (0.135 h^{-1}). Even though benzoate and *p*-ethylphenol consumption rates (0.12 mM h^{-1} and 0.08 mM h^{-1}, respectively) were highly similar to the wild type, *p*-ethylphenol was depleted much faster than in the wild type (>20 h; wild type: 35 h) accompanied by a lower benzoate consumption (1.8 mM; wild type: 2.4 mM). Similar to the result obtained for growth with the mixture of benzoate and *p*-hydroxyacetophenone (Figure 2d, section "The Δ*etpR* mutant cannot grow with *p*-ethylphenol and *p*-hydroxyacetophenone"), the *etpR*-complemented mutant focuses on *p*-ethylphenol degradation. However, in contrast to the highest growth rate for the benzoate/*p*-ethylphenol mixture, the growth rate for the benzoate/*p*-hydroxyacetophenone mixture was the slowest in case of the *etpR*-complemented mutant. The different physiological results may be attributed to the generally different growth rates and lag-phases of the wild type and the *etpR*-complemented mutant provided with *p*-ethylphenol and *p*-hydroxyacetophenone as single substrates (Figure 2) (**a**). Proteomic analysis of wild type, Δ*etpR* mutant and *etpR*-complemented mutant grown with the mixture of benzoate and *p*-ethylphenol. Mascot scores of identified proteins indicated by the intensity of grey shading (lowest Mascot score 71; highest Mascot score 1548). *p*-Ethylphenol-specific proteins were identified in case of the wild type and *etpR*-complemented mutant, but not for the Δ*etpR* mutant. This is in accord with identified transcripts and proteins for the benzoate/*p*-hydroxyacetophenone conditions (see Figure 3), demonstrating the requirement of EtpR and either *p*-ethylphenol or *p*-hydroxyacetophenone as effectors to initiate expression of the "*p*-ethylphenol" gene cluster (**b**). Abbreviations in figure: *ΔetpR* mutant: *ΔetpR*; *etpR*-complemented mutant: *etpR*-compl..

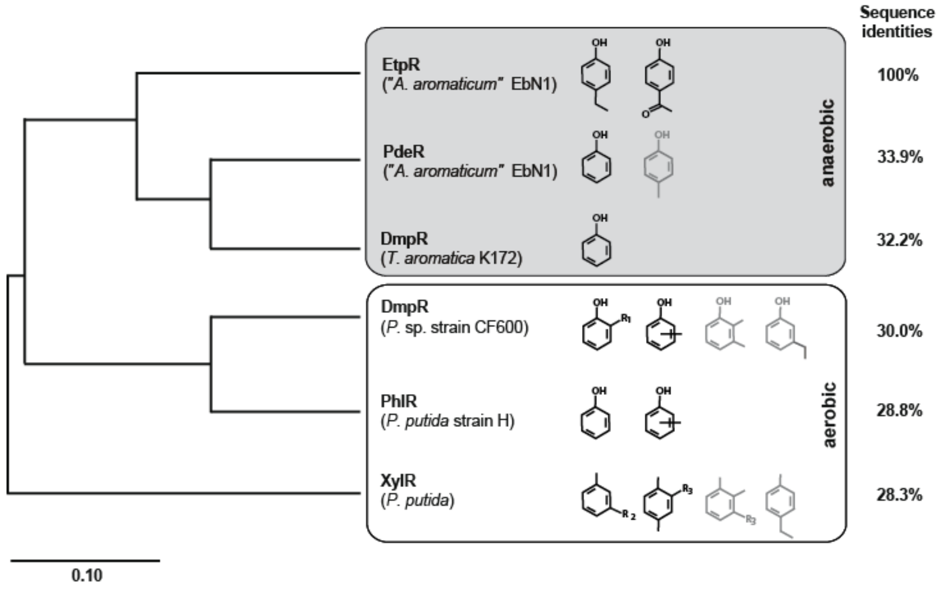

Figure S4 Phylogenetic relations of regulatory proteins. Phylogenetic relationship of the regulatory domains of σ^{54}-dependent NtrC-type regulators EtpR ("*A. aromaticum*" EbN1), PdeR ("*A. aromaticum*" EbN1), DmpR (*T. aromatica* K172), DmpR (*P. putida* CF600), PhlR (*P. putida* strain H) and XylR (*P. putida*) (accession numbers are as given in legend to Figure S5 below). A maximum likelihood tree was generated using ARB (version 5.3): After aligning the regulatory domain sequences applying the ClustalW method, calculations were conducted with the RaxML (version 7.03) and the "rapid bootstrap analysis" algorithm (500 iterations). The scale bar indicates that 0.1 of the branch length represents 10% estimated amino acid sequence divergence. Sequence identities (compared to EtpR) are calculated based on the aligned amino acid sequences of the regulator domains using the Clustal Omega program provided on the Uniprot web-page (http://www.uniprot.org/align/). Substrates that initiate transcription and serve as growth substrates are depicted in black; aromatic effectors only capable of activating transcription of the respective gene clusters (i.e., gratuitous inducers) are shown in grey (8, 14, 31, 36, 37, 52). For *Pseudomonas* spp. DmpR and XylR selected effectors are given. R_1 = -H, -CH_2CH_3; R_2 = -H, -CH_3, -CH_2CH_3; R_3 = -H, -CH_3.

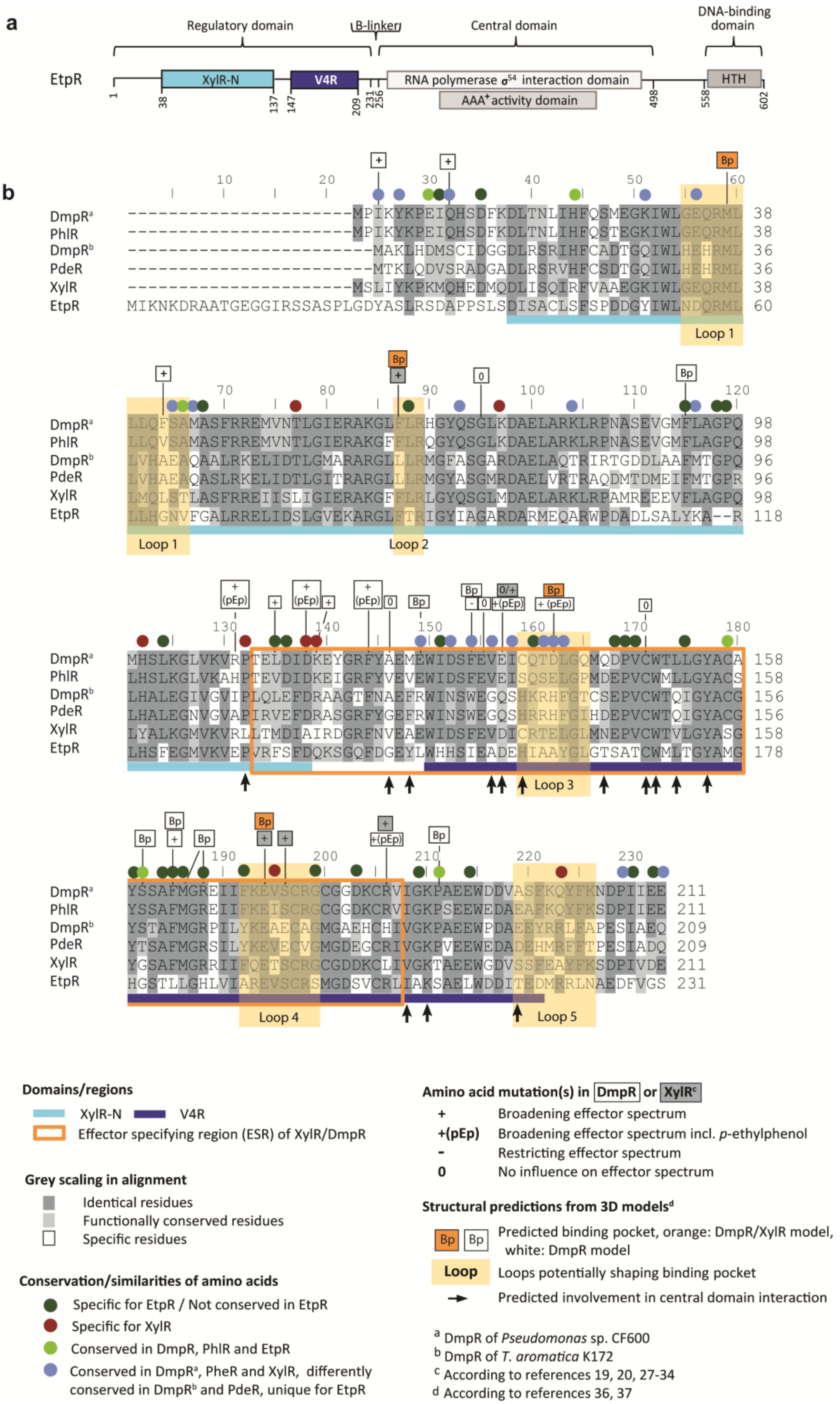
a
Regulatory domain
B-linker
Central domain
DNA-binding domain
EtpR
XylR-N
V4R
RNA polymerase σ54 interaction domain
AAA+ activity domain
HTH
1
38
137
147
209
231
256
498
558
602
b
DmpRa
PhlR
DmpRb
PdeR
XylR
EtpR
Loop 1
Loop 2
Loop 3
Loop 4
Loop 5
Bp
+
0
-
+(pEp)
0/+
Domains/regions
XylR-N
V4R
Effector specifying region (ESR) of XylR/DmpR
Grey scaling in alignment
Identical residues
Functionally conserved residues
Specific residues
Conservation/similarities of amino acids
Specific for EtpR / Not conserved in EtpR
Specific for XylR
Conserved in DmpR, PhlR and EtpR
Conserved in DmpRa, PheR and XylR, differently conserved in DmpRb and PdeR, unique for EtpR
Amino acid mutation(s) in DmpR or XylRc
+ Broadening effector spectrum
+(pEp) Broadening effector spectrum incl. p-ethylphenol
- Restricting effector spectrum
0 No influence on effector spectrum
Structural predictions from 3D modelsd
Bp Bp Predicted binding pocket, orange: DmpR/XylR model, white: DmpR model
Loop Loops potentially shaping binding pocket
Predicted involvement in central domain interaction
a DmpR of Pseudomonas sp. CF600
b DmpR of T. aromatica K172
c According to references 19, 20, 27-34
d According to references 36, 37

Figure S5 Full length EtpR regulatory domain alignment. Amino acid sequence comparison of σ^{54}-dependent regulators involved in aromatic compound catabolism. Domain structure of EtpR: XylR-N, activator of aromatic catabolism (IPR010523); V4R, 4-vinyl reductase (IPR004096); RNA polymerase σ^{54} interaction domain (IPR002078); AAA+ ATPase domain (IPR003593); DNA-binding HTH domain, Fis-type (IPR002197) (**a**). Alignment of the complete regulatory domains of *Pseudomonas* spp. DmpR (YP_009074430), PhlR (YP_009074867) and XylR (YP_009074187) as well as *T. aromatica* K172 DmpR (CAC12684), and "*A. aromaticum*" EbN1 PdeR (CAI07889) and EtpR (CAI06292) (**b**).
